# Genetic basis of latitudinal variation in vertebral number in the *Oryzias latipes* species complex

**DOI:** 10.1186/s40851-025-00256-1

**Published:** 2025-12-05

**Authors:** Rie Hara, Satoshi Ansai, Yasuhiro Kamei, Masaru Matsuda, Masato Kinoshita

**Affiliations:** 1 Division of Applied Biosciences, Graduate School of Agriculture, Kyoto University, Kitashirakawa-Oiwake-cho, Sakyo-ku, Kyoto, 606-8502 Japan; 2 Ushimado Marine Institute, Okayama University, Okayama, 701-4303 Japan; 3 The Graduate University for Advanced Studies (SOKENDAI), Myodaiji Nishigo-naka 38, Okazaki, Aichi 444-8585 Japan; 4 Optics and Imaging Facility, Trans-Scale Biology Center, National Institute for Basic Biology, Myodaiji Nishigo-naka 38, Okazaki, Aichi 444-8585 Japan; 5 Center for Bioscience Research and Education, Utsunomiya University, Utsunomiya, 321-8505 Japan

**Keywords:** Fish, Local adaptation, Meristic variation, Bioresource, Evolution, GWAS, micro-CT

## Abstract

Latitudinal variation plays an important role in driving local adaptation and intraspecific polymorphism across a wide range of organisms. Among teleost fishes, populations from higher latitude tend to have more vertebrae than those from lower latitude, which is known as “Jordan’s rule”. Vertebral number is determined during embryogenesis by the number of somites formed, but the genetic mechanisms underlying this latitudinal variation remain largely unclear. The medaka (*Oryzias latipes*) species complex, including *O. latipes*, *O. sakaizumii*, *O. sinensis* and an undescribed species from East Korea, is a freshwater teleost species group distributed across a latitudinal range from 25° to 40°N. This wide geographical distribution makes medaka an ideal model system for studying latitudinal variation in morphological trait. In this study, we found a positive correlation between abdominal vertebral number and the latitude of original sampling sites of 90 wild-derived stocks of the *O. latipes* species complex. To further examine the environmental and genetic contributions to this trait, we reared 10 selected stocks originating from different latitudes at four incubation temperatures (22, 24, 26, 28 °C) until somite stage. Across all temperatures within each phylogenetic subgroup, stocks from higher latitudes consistently developed a greater number of abdominal vertebrae. Finally, we performed a genome-wide association study (GWAS) to identify genomic loci associated with vertebral number variation in these wild-derived stocks. This analysis revealed a single nucleotide polymorphism (SNP) on chromosome 10 that was significantly associated with total vertebral count. Taken together, our findings demonstrate that wild-derived stocks of the *O. latipes* species complex retain latitudinal variation in vertebral number. This system provides a powerful framework for investigating the evolutionary and genetic bases of geographic trait variation in teleost fishes.

**Supplementary Information:**

The online version contains supplementary material available at 10.1186/s40851-025-00256-1.

## Background

Latitudinal variation influences annual patterns of temperature and photoperiod, which can drive local adaptation in many organisms. Such variations play a crucial role in shaping trait evolution and have been linked to intraspecific polymorphism. In endothermic animals, for instance, Bergmann’s rule [1] and Allen’s rule [2] explain how latitude-related temperature difference influence body size and surface area. Similarly, human skin color is correlated with latitude as an adaptation to ultraviolet radiation intensity particularly in low-latitude regions [3].

Among teleost fishes, ectothermic vertebrate inhabiting aquatic environments, latitudinal adaptation has been well-documented in terms of photoperiod, reproductive timing, and thermal tolerance [4, 5]. For example, Atlantic silversides (*Menidia menidia*) at higher latitudes experience shorter growing seasons with optimal temperatures, leading to accelerated growth rates [6, 7]. Also, for adaptation to shorter spawning seasons, reef fish (*Pomacentrus coelestis*) in higher latitude regions have evolved higher egg production efficiency [8]. In contrast, killifish (*Fundulus heteroclitus*) in lower-latitude regions exhibit greater thermal tolerance due to changes in heat shock protein expression [9].

Among the traits exhibiting latitudinal variations in teleosts, vertebrae morphology stands out due to its consistent latitudinal patterning. Jordan’s rule posits that fish populations from higher latitude tend to have more vertebrae than those from lower latitude [10]. While the ecological significance of this pattern has not been fully elucidated, some hypotheses are presented. For example, medaka from higher latitude needs to have more growing capacity to adapt the short growing season [11–13], and previous study actually showed that *O. sakaizumii* from higher latitude had longer guts compared to *O. latipes* [14], thus follows Jordan’s rule in abdominal vertebral bone number [15]. Also, since water temperature affects water viscosity that affects aquatic locomotion [16], vertebral number variation may function to regulate the appropriate swimming ability for fish larvae [17, 18]. However, the underlying genetic and molecular mechanisms of Jordan’s rule remain poorly understood.

**Fig. 1:**
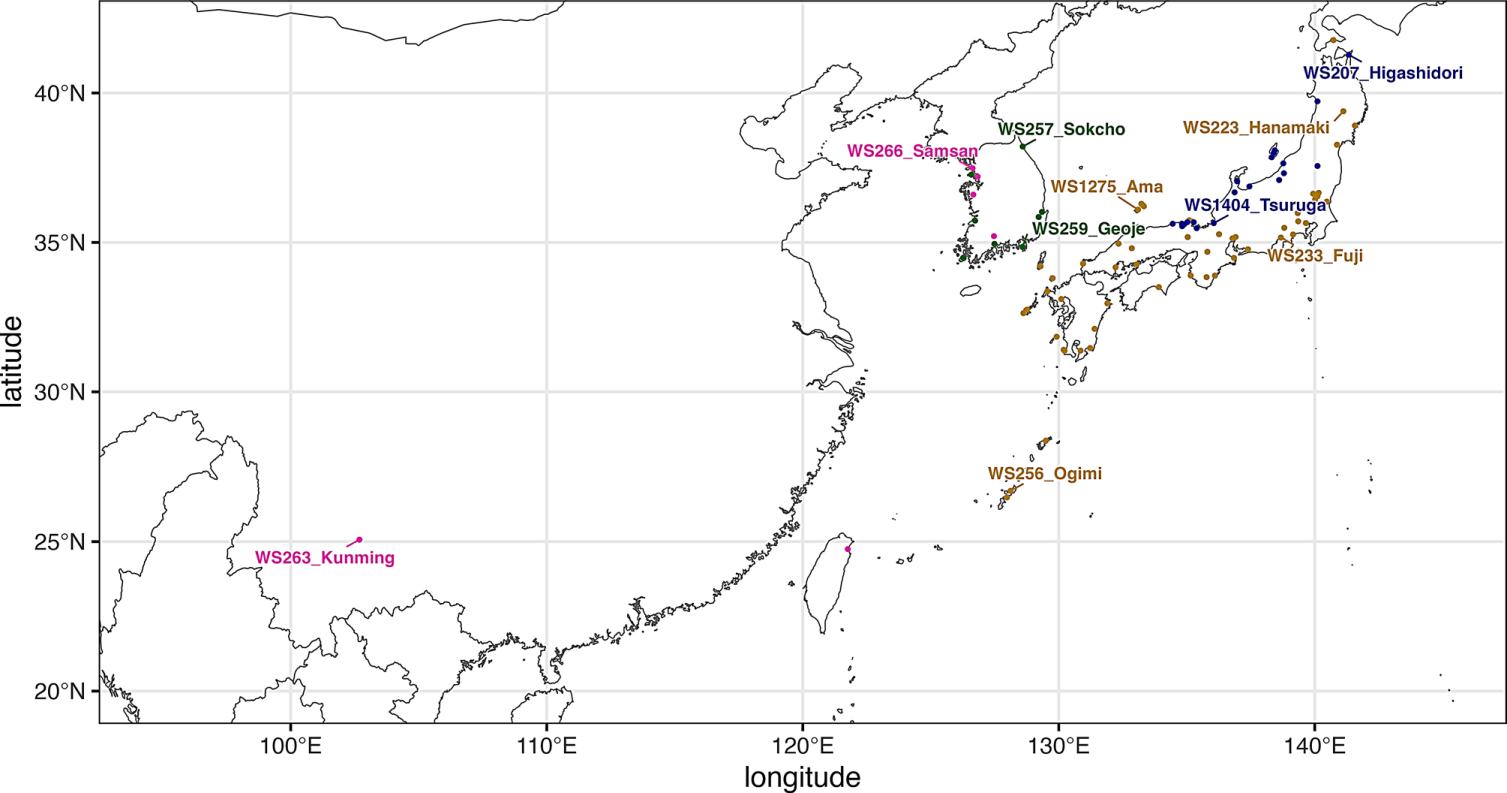
A map showing the original sampling locations of wild-derived medaka stocks. Different dot color indicates different species; blue: *Oryzias sakaizumii*, yellow: *O. latipes*, green: *Oryzias* sp. (East Korean), pink: *O. sinensis*. The sites highlighted with the name indicate 10 stocks used for laboratory rearing experiment. The geographic positions of all sites are shown in Supplementary Figure S1 and Supplementary Table S2

The medaka (*Oryzias latipes*) complex—comprising *O. latipes* from southern Japan, *O. sakaizumii* from northern Japan, *O. sinensis* from western Korea and China, and an undescribed species (*Oryzias sp.*) from East Korea—is a freshwater teleost species complex that spans latitudes from 25° to 40°N [19] (Fig. 1; Supplementary Fig. S1). This geographical range makes the group a valuable model for studying latitudinal variation in growth rate [11–13], reproductive cycles [5], and sexual dimorphism [20]. Previous studies have provided genetic evidence supporting Jordan’s rule in wild populations of *O. sakaizumii*, specifically with respect to the number of abdominal vertebrae [15, 21]. However, the molecular genetic basis of this variation remains unknown, and it is still unclear whether the broader *O. latipes* complex consistently exhibits this pattern across its range.

The National BioResource Project (NBRP) Medaka (Okazaki, Japan) has maintained wild-derived medaka stocks as closed colonies in outdoor fields since 1985 [22]. These wild stocks (more than 90 strains) were originally collected from sites across a broad latitudinal gradient in East Asia (Fig. 1). The stocks have been maintained under common garden conditions, minimizing environmental variability while preserving intrinsic genetic diversity. Recently, whole-genome sequencing of these stocks has been completed, and the SNP data are now available through MedakaBase [23]. This resource allows for both forward and reverse genetic approaches to investigating the molecular basis of phenotypic variation. Given these advantages, these wild-derived medaka stocks represent a powerful model system for studying the evolutionary mechanisms driving latitudinal variation in vertebral traits in teleost fishes.

In this study, we investigated whether vertebral number in the *O. latipes* complex conforms to Jordan’s rule. We quantified vertebral number across wild-derived medaka stocks and used a phylogenetically corrected statistical approach to assess latitudinal trends. Because the fish stocks were reared in outside environments subject to fluctuating water temperatures, we also examined the effect of developmental temperature on vertebral count, building on previous findings that temperature influences vertebral development in medaka [15, 24, 25]. Finally, we conducted a genome-wide association study (GWAS) to identify genomic regions associated with vertebral number variation in these wild-derived stocks.

## Materials and methods

### Vertebral count of wild-derived stocks reared in an outside field

Wild-derived stocks of medaka were obtained from NBRP Medaka. These stocks are maintained at an outside field of Utsunomiya University (Utsunomiya, Japan). On June 12-13th and 26-27th in 2023, adult fish (6 or more months old) of 3 males and 3 females were randomly sampled for each strain. Sex and age of each sample are available with Strain ID in Supplementary Table S1. The fish were fixed with 100% ethanol and preserved at -20℃ for the following analysis.

Vertebral column was imaged by X-ray computed tomography (CT) scanning. Using a three-dimensional micro-CT instrument (R_mCT2; Rigaku, Akishima, Japan), samples were scanned at 90 kV tube voltage and 160 µA tube current. Each sample was scanned twice to separately image for the abdominal and caudal parts with a 20 mm field of view. Samples were rotated 360° in steps of 3° over 2 min, and images of 512 × 512 pixels were generated. Three-dimensional image was reconstructed using Horos software (version 3.3.6; Horos Project) to count vertebral number. The first caudal vertebra was defined as the first vertebra with a fused haemal spine and located just behind the insertion of the anal pterygiophore (Supplementary Fig. S2). Also, the second preural centrum was defined as the last caudal vertebra. The definitions of abdominal vertebrae and total number of vertebrae were followed by a previous study (Supplementary Fig. S2) [15].

### Vertebral count of laboratory-raised fish under controlled temperature

For laboratory experiments under controlled temperature, we used the following 10 wild-derived stocks: Higashidori (NBRP ID: WS207), Hanamaki (ID: WS223), Fuji (ID: WS233), Ogimi (ID: WS256), Sokcho (ID: WS257), Geoje (ID: WS259), Samsan (ID: WS266), Ama (ID: WS1275), Tsuruga (ID: WS1404), and Kunming (ID: WS263).

Early embryos at stage 10 or earlier [26] were collected from each stock. Subsequently, 3 to 25 eggs were incubated in 10 mL of reverse osmotic water with methylene blue at four temperatures: 22, 24, 26, 28 ± 0.1℃. A previous study showed that rearing density of fertilized eggs (25 to 200 eggs per 100mL of water) did not affect the number of vertebral bones in medaka [25]. Once the embryos reached stage 33 (somite completion stage is stage 32), they were transferred to 26 ± 0.1℃ until hatching to reduce mortality associated with prolonged exposure to extreme temperatures. After hatching, larvae were maintained under a 14/10-h light/dark cycle at 26 °C and fed newly hatched brine shrimp nauplii (Marinetech, Tahara, Japan) and dry feed (Ayu Super Gold 0; Marubeni Nisshin Feed Co., Tokyo, Japan). Fish reached stage 43 (over 10.0 mm TL) were fixed in 100% ethanol and stained overnight with Alizarin red S solution. Stained samples were bleached overnight with 0.075% H_2_O_2_ and 0.5% KOH. Finally, specimens were imaged in a mixture of 50% glycerol and 0.25% KOH. Using the captured images, we counted abdominal, caudal, and total vertebral numbers in each specimen.

For statistical analysis, we analyzed the vertebral counts using nonparametric factorial analysis of variance (ANOVA) with Aligned Rank Transform (ART-ANOVA) using the R package ‘ARTool’. Multiple comparison with Tukey method regarding each fixed effect was examined based on estimated marginal means obtained by the R package ‘emmeans’.

### Whole-genome sequencing and phylogenomics in wild-derived stocks

Whole-genome sequencing data were obtained for 94 wild-derived medaka stocks from a public repository (NCBI BioProject: PRJNA1298385). For each stock, genome DNA was extracted from a single male individual and sequenced using DNBSEQ platforms (2 × 150 bp paired-end reads) at BGI Japan (Kobe, Japan). On average, 41.93 ± 8.58 Gb of sequencing data were generated per individual. Raw reads were trimmed using *fastp* v0.23.3 and aligned to the *O. latipes* Hd-rR reference assembly (ASM223467v1) [27] using *bwa-mem2* v2.2.1. Alignments were sorted by coordinate, and PCR duplicates were removed using *elprep* v5.1.3. Variant calling was performed on uniquely mapped reads (MQ >10) using *bcftools* v1.17 (*mpileup* and *call*). Variants were filtered to retain only those with sufficient read depth (DP >10), high variant quality (QUAL >30), high genotype quality (GQ >30), and present in more than 90% of individuals using *VCFtools* v0.1.16.

To infer the phylogenetic relationship among the wild-derived stocks, we identified the coding sequences of 3,618 single-copy orthologues across Actinopterygii based on OrthoDBv10 (actinopterygii_db10) using BUSCO v5.8.0. From these, 3,402 complete single-copy BUSCOs were recovered and aligned, resulting in a concatenated alignment of 5,717,647 bp. Each gene was treated as a separate partition, and a maximum likelihood phylogenetic tree was constructed using *IQ-TREE* v2.3.0.

### Association between latitude and vertebral number

To examine whether medaka stocks originated from higher latitude have more vertebrae, we statistically tested the relationship between latitude of the original sampling sites and vertebral number using a phylogenetic generalized least squares (PGLS) model. In this framework, regression coefficients are estimated while accounting for phylogenetic non-independence by incorporating a covariance matrix derived from the topology and branch lengths of a phylogenetic tree. We constructed PGLS models using the ‘pgls’ function in the R package *caper* [28]. The geographic coordinates of the original sampling site of each stock were defined based on the city or town hall associated with the locality (Supplementary Table S2). Separate PGLS models were constructed using latitude as the explanatory variable for the log-transformed counts of abdominal, caudal, and total vertebrae. Branch lengths were optimized using maximum likelihood estimation (“ML” for the ‘lambda’ option in the ‘pgls’ function). To assess the relative contributions of latitude and phylogeny, we calculated partial coefficients of determination (partial R^2^) using the ‘R2’ function in the R package *rr2* [29]. Specifically, we used the R^2^_lik_ method [30] to estimate partial R^2^. Since the ‘R2’ function does not support ‘pgls’ objects directly, we reimplemented the models using ‘phylolm’ function from the *phylolm* package for this analysis.

### Genome-wide association study (GWAS)

Genome-wide association analyses were conducted to identify genomic loci associated with variation in vertebral traits across 76 stocks consisting of *O. latipes* and *O. sakaizumii*. Association testing was conducted using GEMMA (v.0.98.3) [31], employing linear mixed models that incorporate a genetic relatedness matrix to control for population structure. Prior to analysis, SNPs were pruned for linkage disequilibrium using PLINK v1.190b6,21 with --indep-pairwise 50 10 0.2 option, and filtered to retain only variants with a minor allele frequency of at least 5%. After filtering, 1,065,978 SNPs were retained in the dataset. Statistical significance of associations was evaluated using the Wald test with Bonferroni correction for multiple comparisons.

## Results

### Correlations between latitude and vertebral number in wild-derived stocks reared in an outside field

**Fig. 2:**
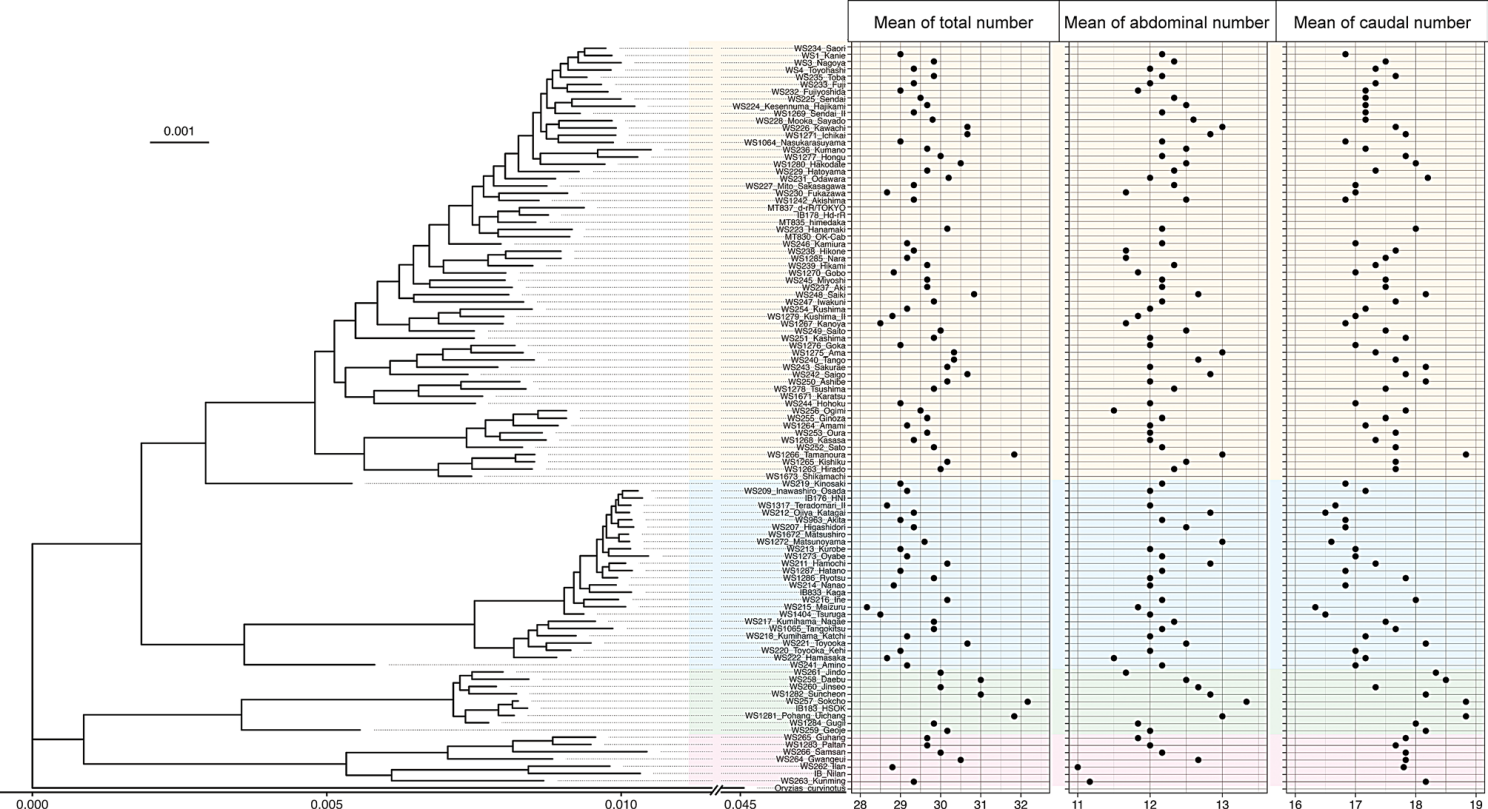
Phylogenetic relationship and vertebral bone numbers of wild-derived medaka stocks. The left panel shows a maximum likelihood phylogenetic tree inferred using coding sequencing of 3,402 single copy BUSCOs. The right panels indicate mean vertebral numbers is mean of 6 individuals (3 females and 3 males) per stocks. Each dot represents mean of total (left), abdominal (middle), and caudal (right) vertebral bone number. The color indicates different medaka species; yellow: *O. latipes*, blue: *O. sakaizumii*, green: *Oryzias* sp. (East Korean) and pink: *O. sinensis*

**Fig. 3:**
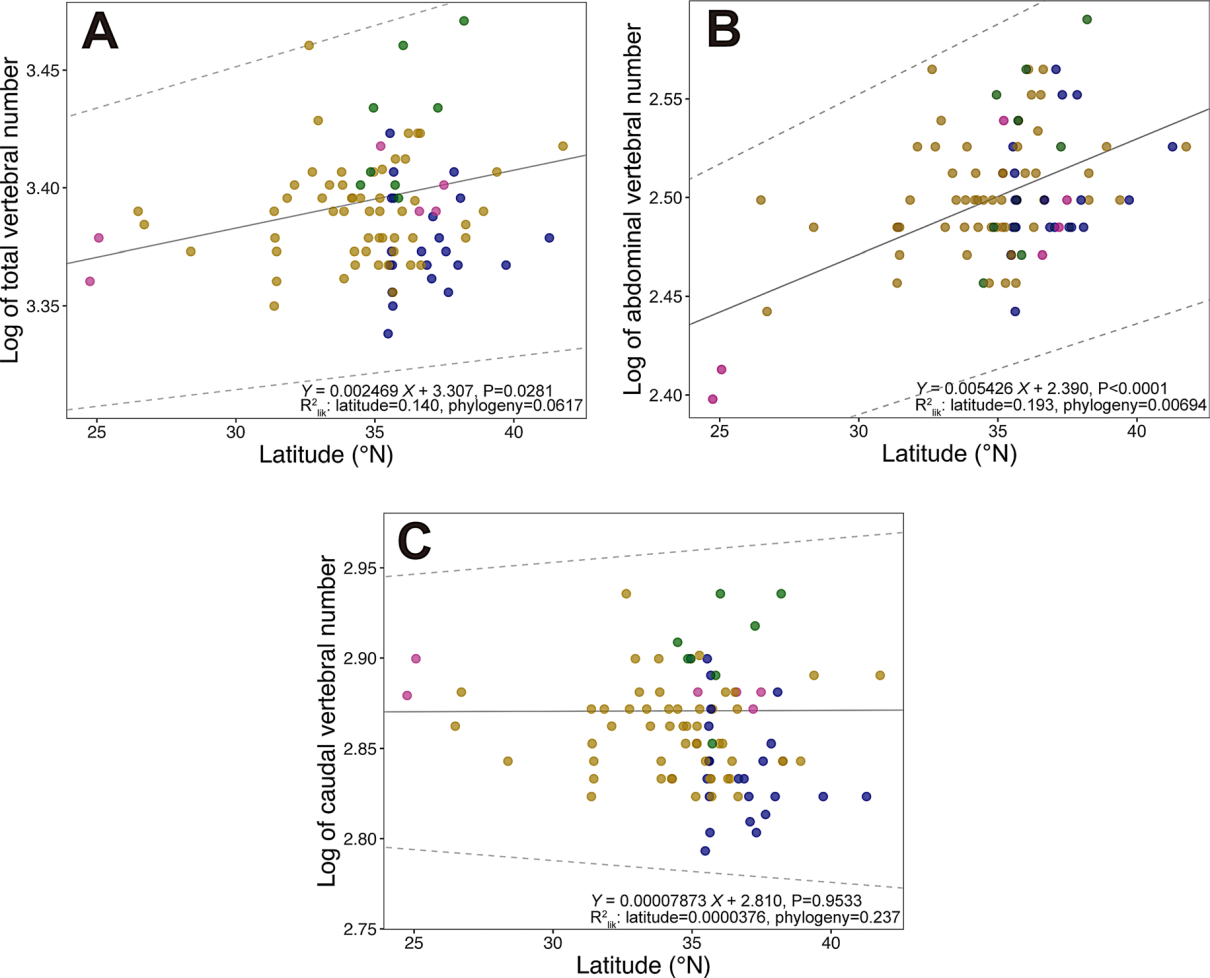
Association between latitude and log10-transformed (**A**) total, (**B**) abdominal, and (**C**) caudal vertebral bone numbers in 90 wild-derived medaka stocks. Latitude of the original sampling site is included as an explanatory variable of phylogenetic generalized least squares (PGLS) analysis. The solid and dashed lines show linear regression from coefficient ± standard error (SE) of each PGLS analysis, respectively. partial R_2_ for latitude and phylogeny are also shown). Each dot represents log-transformed mean vertebral number of each stock. The different dot color indicates different medaka species; blue: *O. sakaizumii*, yellow: *O. latipes*, green: *Oryzias* sp. (East Korean), pink: *O. sinensis*

To test the hypothesis that medaka populations derived from higher latitude have more vertebral bones, we analyzed 90 wild-derived medaka stocks that were originally derived from East Asia (Fig. 1). First, phylogenomic analysis based on coding sequences of 3,402 single-copy BUSCOs demonstrated that the wild-derived stocks consist of 4 genetically distinct clades (Fig. 2) corresponding to 4 different species of *O. latipes* complex including *O. latipes*, *O. sakaizumii*, *O. sinensis*, and *Oryzias* sp. (East Korean) [19], similar to a previous study inferred using allozyme [32] and genome-wide single nucleotide polymorphisms (SNPs) [33]. Then, abdominal and caudal vertebral bone numbers of 6 adult fish were counted using micro-CT images in each stock. Statistical analysis with PGLS revealed that latitude of original sampling sites of the stocks showed positive correlation with abdominal vertebral number but not with caudal and total vertebral numbers (Fig. 3; Table 1). Correlation coefficients of the PGLS models computed as Partial R² showed that the latitude explained a larger proportion of the variance in the abdominal vertebral number (R^2^_lik_ = 0.191) compared to the phylogenetic structure (R^2^_lik_ = 0.00937) (Fig. 3B). In contrast, the phylogenetic structure explained a larger proportion of the variance in the caudal vertebral number (R^2^_lik_ = 0.225) compared to latitude (R^2^_lik_ = 1.82e-05) (Fig. 3C) as well as the total vertebral number (latitude R^2^_lik_ = 0.0589; phylogeny R^2^_lik_ = 0.185) (Fig. 3A).

**Table 1:**
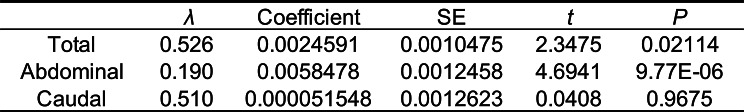
Phylogenetic generalized least squares (PGLS) analysis of vertebral bone numbers in wild-derived Medaka stocks. The models include latitude of the original sampling site as explanatory variable and log10-transformed total, abdominal, and caudal vertebral bone numbers as response variables

### Correlations between latitude and vertebral number in controlled temperature fish

**Fig. 4:**
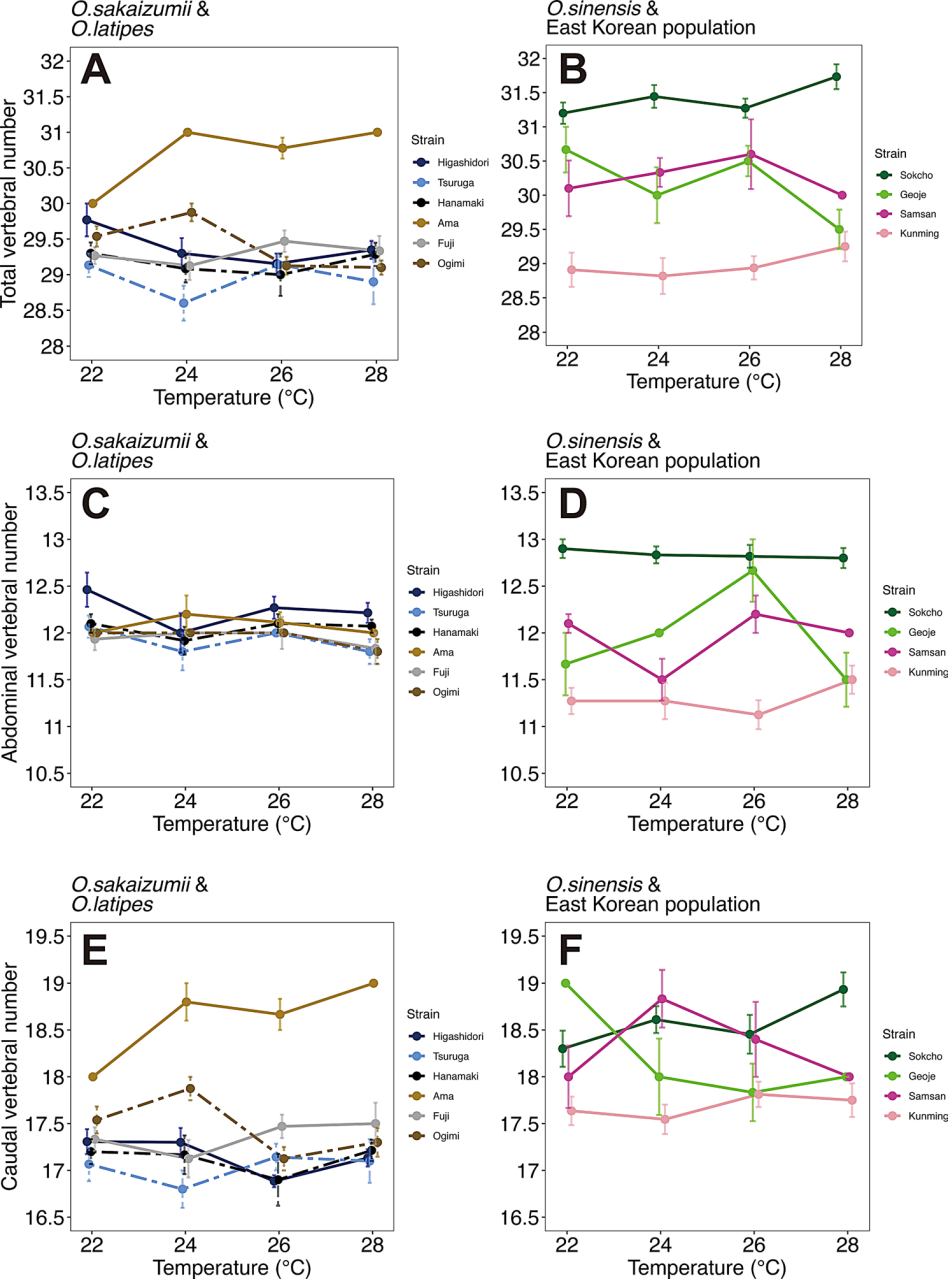
Reaction norms of total (**A**, **B**), abdominal (**C**, **D**), and caudal (**E**, **F**) vertebral bone number in 10 wild-derived stocks reared at different water temperatures. The left panels (**A**, **C**, **E**) indicate *Oryzias sakaizumii*, Higashidori (NBRP ID: WS207) and Tsuruga (ID: WS1404), and *O. latipes*, Hanamaki (ID: WS223), Fuji (ID: WS233), Ogimi (ID: WS256), and Ama (ID: WS1275), while the right ones (**B**, **D**, **F**) show *Oryzias* sp. (East Korean), Sokcho (ID: WS257) and Geoje (ID: WS259), and *O. sinensis*, Samsan (ID: WS266) and Kunming (ID: WS263). Each dot and error bar represent mean and standard error of vertebral bone number, respectively. Different colors indicate different stocks. Vertebral numbers of each analyzed individual are shown in Supplementary Figure S3

Wild-derived stocks of medaka analyzed in this study were separately raised and maintained in an outside field, and thus they were likely to experience different water temperature. Importantly, previous studies demonstrated that the experienced water temperature, especially during their developmental stages, affects vertebral bone numbers of medaka [15, 24, 25]. To investigate the effects of water temperature during the development on vertebral bone numbers, fertilized eggs obtained from pure crosses of wild-derived stocks were developed at 4 different water temperatures (22, 24, 26, 28 ˚C) and then raised to the young-adult stage. Among the 90 stocks analyzed in this study, we chose 10 stocks that were originally derived from sampling sites with various latitudes from 4 different species: Higashidori, Hanamaki, Tsuruga (*O. sakaizumii*), Fuji, Ama, Ogimi (*O. latipes*), Sokcho, Geoje (East Korean), Samsan and Kunming (*O. sinensis*) (Figs. 1 and 2). Even in the laboratory condition, stocks from higher latitude tended to have more abdominal vertebrae at any temperature in each species (Fig. 4C and D). Especially, Sokcho (*Oryzias* sp. East Korea) showed the most (median = 13) of abdominal vertebrae while Kunming (*O. sinensis*) showed the least (median = 11) despite the temperature change (Fig. 4D). Aligned rank transform-analysis of variance (ART-ANOVA) including stocks and water temperature as explanatory variables for the vertebral number revealed that genetic difference (strain) significantly affected all the abdominal, caudal and total vertebral number whereas water temperature did not significantly affect the vertebrae numbers except the caudal vertebrae (Table 2). Multiple comparison of caudal vertebral counts at different temperatures showed that caudal vertebrae were significantly decreased at 26 ˚C but not at 28 ˚C (Supplementary Table S3). Unlike the previous study in *Oryzias sakaizumii* strains [15], no clear increasing trend was observed in fish reared at lower temperatures (Fig. 4). Also, significant interactions between strain and water temperature were observed in the caudal and total number of vertebrae (Table 2), which could be caused by increased total and caudal vertebral counts at higher temperature observed in the Ama strain (Fig. 4A and E).

**Table 2:**
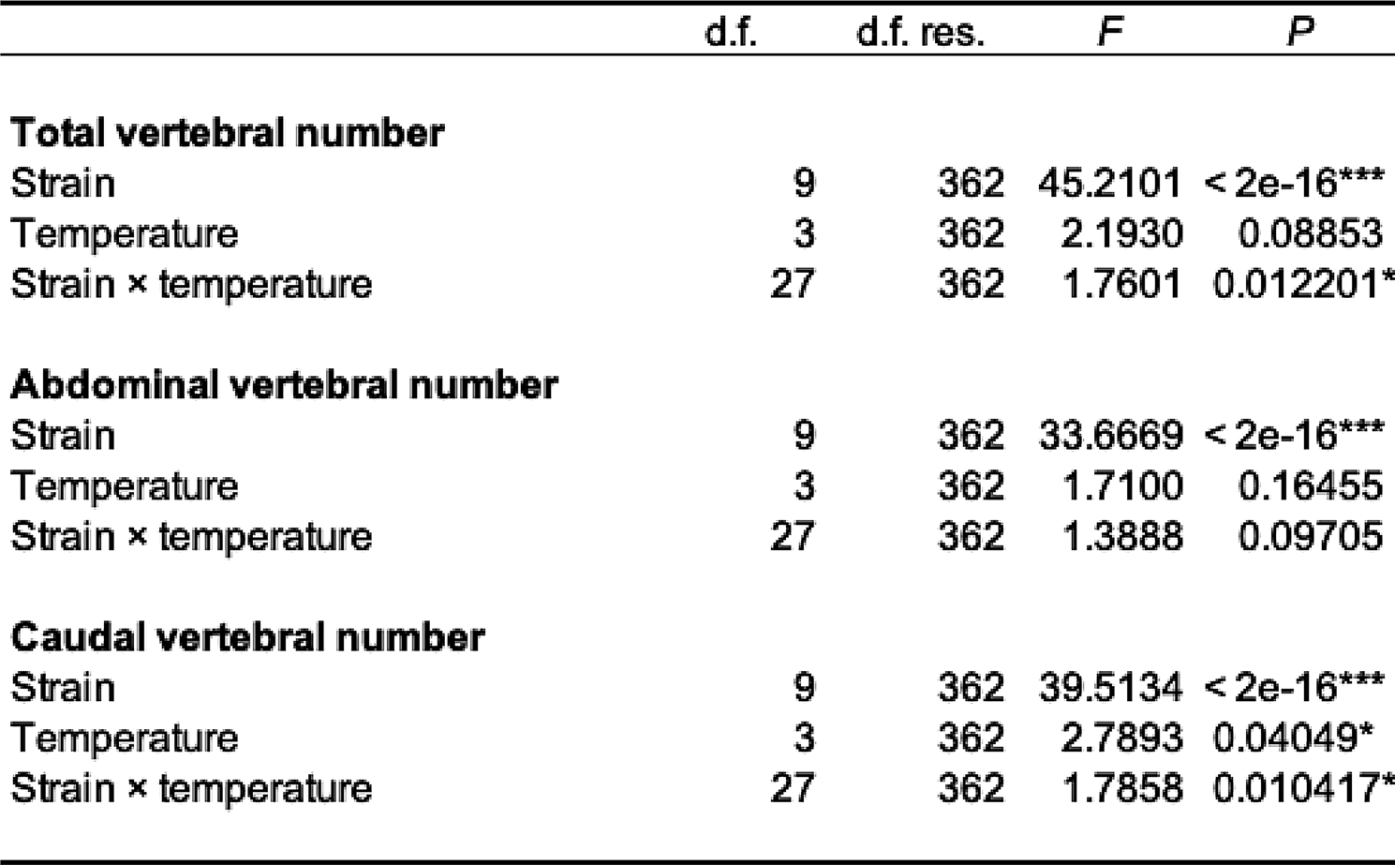
Results of aligned rank transform-analysis of variance (ART-ANOVA) in vertebral numbers of laboratory-raised fish. The effects of strain and water temperature on the vertebral bone numbers of total (A), abdominal (B), and caudal (C) were examined. *P < 0.05 and ***P < 0.001

### GWAS mapping for vertebral number traits

**Fig. 5:**
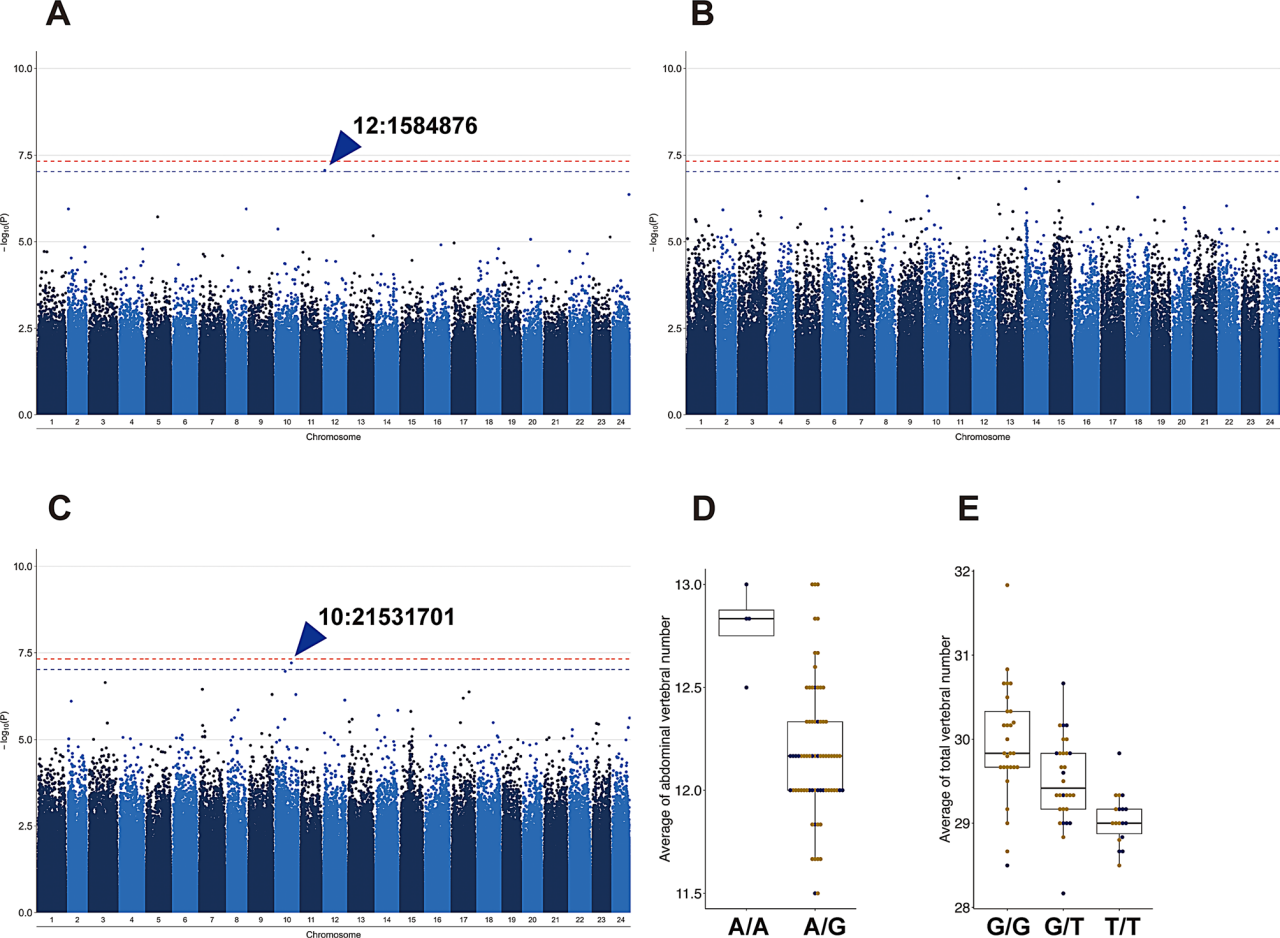
Genome-wide association mapping of vertebrae number in 76 stocks consisting of *O. latipes* and *O. sakaizumii*. Manhattan plots showing the significance of SNPs associated with the abdominal (**A**), caudal (**B**), and total (**C**) number of vertebrae. The blue and red dashed horizontal lines indicate the significance thresholds for the Wald test with Bonferroni correction, each line indicating *p* = 0.1 and 0.05. (**D**, **E**) The genotype of the significant SNP is shown in relation to the abdominal vertebral number (**D**) and total vertebral number (**E**) of *O. latipes* (yellow) and *O. sakaizumii* (blue) stocks included in the analysis

To identify genomic regions associated with variations in vertebral number, genome-wide association mapping was conducted (Fig. 5). Since East Korean populations and *O. sinensis* are genetically distant from *O. latipes* and *O. sakaizumii* (Fig. 2), these two populations were excluded from the GWAS mapping to avoid confounding from population structure. In the *O. latipes* and *O. sakaizumii* subset, analysis of abdominal vertebral number revealed one significant SNP on chromosome 12 at 1.58 Mb position (Fig. 5A and D), and also analysis of total vertebral number in *O. latipes* and *O. sakaizumii* populations revealed one significant SNP on chromosome 10 at 21.5 Mb position (Fig. 5C and E). The proportion of variance in phenotypes explained (PVE) indicating heritability of traits was calculated by GEMMA, that were 0.16 ± 0.28, 0.24 ± 0.16, and 0.29 ± 0.21 for abdominal, caudal, and total vertebral number traits.

## Discussion

In this study, we quantified vertebral numbers in 90 wild-derived medaka stocks and found that fish from higher-latitude populations exhibited significantly more abdominal vertebrae. This observation aligns with a previous study that reported a similar genetic correlation between abdominal vertebral number and habitat latitude in 13 wild populations of *O. sakaizumii* [15]. Together, these findings suggest that abdominal vertebral number of the *O. latipes* species complex follows Jordan’s rule. Notably, since all fish were reared under common garden conditions, the observed latitudinal pattern likely reflects underlying genetic differences rather than environmental effects. These results underscore the value of wild-derived medaka stocks as a model system for investigating the evolutionary and genetic mechanisms underlying vertebral variation in teleosts.

One hypothesis for increased abdominal vertebral number at higher latitudes posits that medaka in these environments require greater growth capacity to compensate for shorter growing seasons [11–13]. Supporting this hypothesis, a previous study reported that *O. sakaizumii* from northern habitats possess longer intestinal tracts than *O. latipes* [14], suggesting that this trait may be conserved across the entire *O. latipes* complex. Our partial R^2^ analysis revealed a significant association between abdominal vertebral number and latitude of the original collection sites, whereas no such correlation was detected for caudal vertebral number. These results are consistent with our PGLS analysis, which also showed a positive correlation between abdominal vertebral number and latitude. Further research is needed to clarify the functional relationship between abdominal vertebrae number and intestinal tract length, and to explore how natural selection acts on variation in abdominal vertebrae within the *O. latipes* species complex.

Previous studies have reported a negative correlation between vertebral number and rearing temperature in medaka [15, 24, 25]. However, in our study using wild-derived stocks reared under controlled conditions, we did not detect a clear temperature-dependent pattern. This discrepancy may be due to several factors. First, the number of individuals per stock in our study was smaller than in previous studies [15, 24, 25], potentially reducing statistical power. Second, the temperature range tested in this study (22–28℃) was narrower than in the earlier studies (20–32℃) [15, 25]. Although vertebral counts at the extreme temperatures (20 and 32˚C) appear to deviate from the weak negative correlation [15, 25], excluding these conditions may obscure temperature effects for other reasons. Third, in this study water temperature was controlled only until the somite stage, whereas in previous studies it was regulated until adulthood. Although vertebral number is generally determined by the number of somites formed during early embryogenesis [34], later developmental stages could still be influenced by water temperature, thereby affecting adult vertebral counts. In teleost fishes, vertebral segmentation is driven by the periodic calcification of the notochordal sheath [35]. This periodicity is influenced by, but independent from, somite segmentation in zebrafish [36, 37]. Although it remains controversial whether periodic calcification is independent of somite segmentation in medaka [38, 39], it is possible that water temperature after somite segmentation affect vertebral number.

To further investigate the genetic basis of vertebral variation, we performed a GWAS using wild-derived stocks of the Japanese species *O. latipes* and *O. sakaizumii*, which identified two SNPs significantly associated with vertebral counts. The SNP on chromosome 12 (12: 1,584,876) showed a significant association with abdominal vertebrae; however, the genotype distribution was highly skewed (Fig. 5D), suggesting a possible statistical artifact. This result implies that factors other than genotype or temperature may primarily contribute to the latitudinal variation in abdominal vertebrae observed in this study. In contrast, a SNP on chromosome 10 (10: 21,531,701) was significantly associated with total vertebral number, with less genotype bias (Fig. 5E), indicating a genetic contribution to total vertebral number.

The GWAS also provided candidate genes potentially underlying latitudinal variations in vertebral number. The SNP on chromosome 12 is located at the 8th intron of the *MARVEL domain-containing protein 2* (*marveld2*), a gene that functions as a tight junction protein and modulates osmoregulation in Atlantic salmon [40]. Although *marveld2* is expressed in medaka presomitic mesoderm [41: E-MTAB-13927 of ArrayExpress], its functional role in somite segmentation has not been examined. No genes with known functions in somite segmentation were identified within ± 100 kb of this locus, although some nearby genes (*bap1*, *tor4a*) are involved in neural Notch signaling [42, 43]. By contrast, the SNP on chromosome 10 lies in an intergenic region with no annotated gene within ± 100 Kb of the SNP. The *teneurin transmembrane protein 2* (*tenm2*) gene is located approximately 193 Kb away; however, neither its functional role in somite segmentation [44] nor its expression in presomitic mesoderm [41: E-MTAB-13927 of ArrayExpress] has been clearly demonstrated. Further investigations of the genomic region surrounding these SNPs may reveal novel genetic factors influencing latitudinal variations in vertebral number in wild populations of *O. latipes* and *O. sakaizumii*.

## Conclusions

In the present study, we found that wild-derived stocks of the *Oryzias latipes* species complex from higher-latitude populations genetically exhibited a greater number of abdominal vertebrae, consistent with Jordan’s rule. This tendency persisted across different developmental temperature. Finally, genome-wide association analysis identified a SNP on chromosome 10 that was significantly associated with total vertebral number in Japanese species *O. latipes* and *O. sakaizumii*. These findings underscore the utility of wild-derived medaka stocks as a valuable model for investigating the evolutionary and genetic bases of vertebral variation in teleost fishes.

## Supplementary Information

Below is the link to the electronic supplementary material.

Supplementary Material 1

Supplementary Material 2: Fig. S1. Maps showing the origin locations of wild-derived medaka with ID information. Different colors indicate different medaka species; blue: *O. sakaizumii* (A), yellow: *O. latipes* (B), green: *Oryzias* sp. (East Korean) (C), pink: *O. sinensis* (C).

Supplementary Material 3: Fig. S2. Definition of abdominal and caudal vertebrae. Arrowhead shows the first and last abdominal (A) and caudal (B) vertebrae. Arrow shows the anal pterygiophore (B). This CT reconstruction represents individual No. 6 from the WS1404_Tsuruga stock (Supplementary Table S1).

Supplementary Material 4: Fig. S3. Vertebral bone numbers of each individual of 10 wild-derived stocks reared in controlled laboratory environments. Each panel indicates different water temperatures during their embryonic development; 22˚C (A), 24˚C (B), 26˚C (C), and 28˚C (D). Top, bottom left, and bottom right panels represents total, abdominal, and caudal vertebral bone numbers, respectively.

## Data Availability

The datasets supporting the conclusions of this article are included within the additional files.”
