# Supplementary figures and images for: Genetic basis of latitudinal variation in vertebral number in the Oryzias latipes species complex
Source: Zoological Lett. 2025 Dec 5;12:1. doi: 10.1186/s40851-025-00256-1 (PMC12798130; doi:10.1186/s40851-025-00256-1)

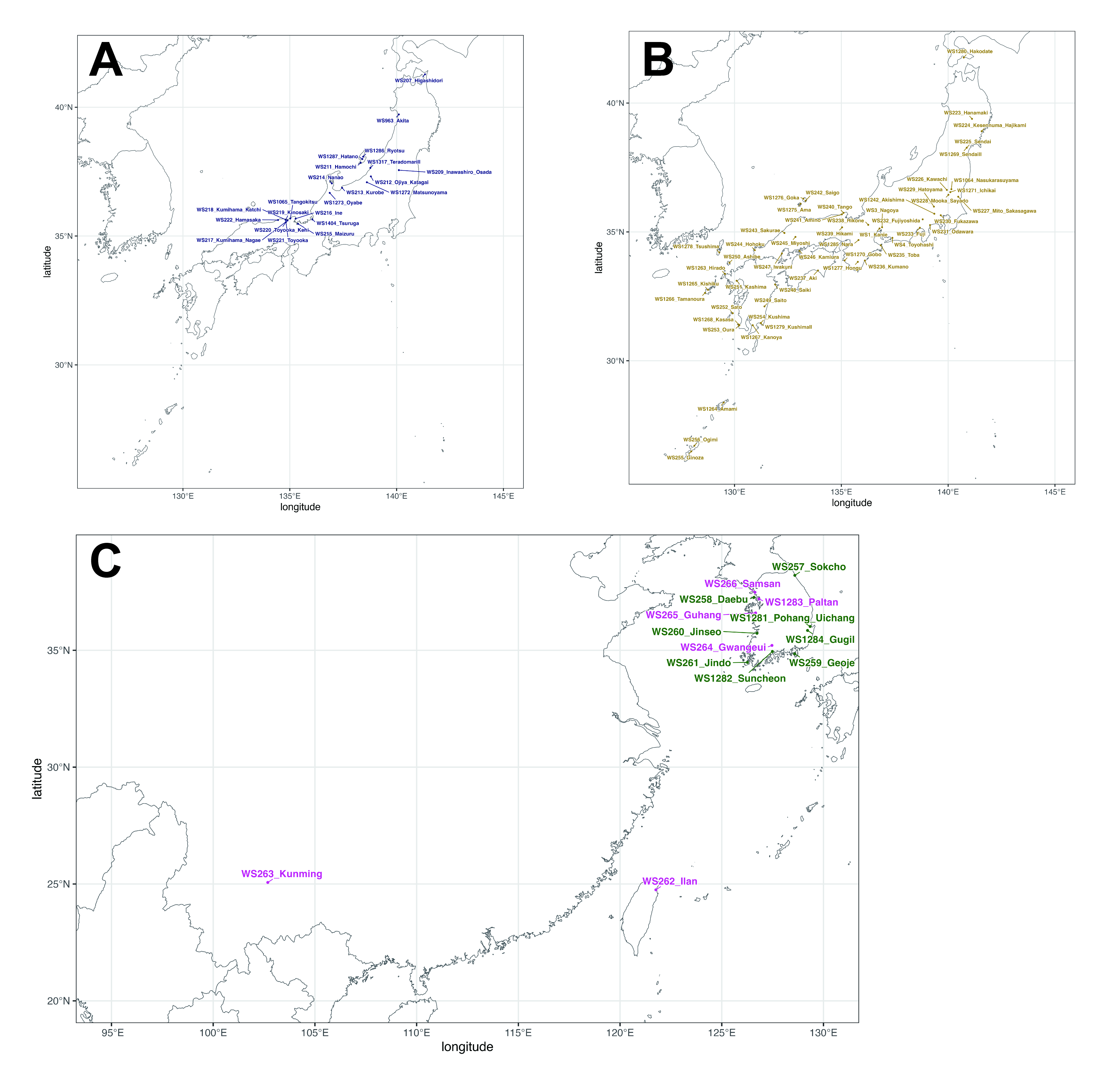

Supplement: Supplementary file 2 — Supplementary Material 2: Fig. S1. Maps showing the origin locations of wild-derived medaka with ID information. Different colors indicate different medaka species; blue: O. sakaizumii (A), yellow: O. latipes (B), green: Oryzias sp. (East Korean) (C), pink: O. sinensis (C). [file 40851_2025_256_MOESM2_ESM.tif]

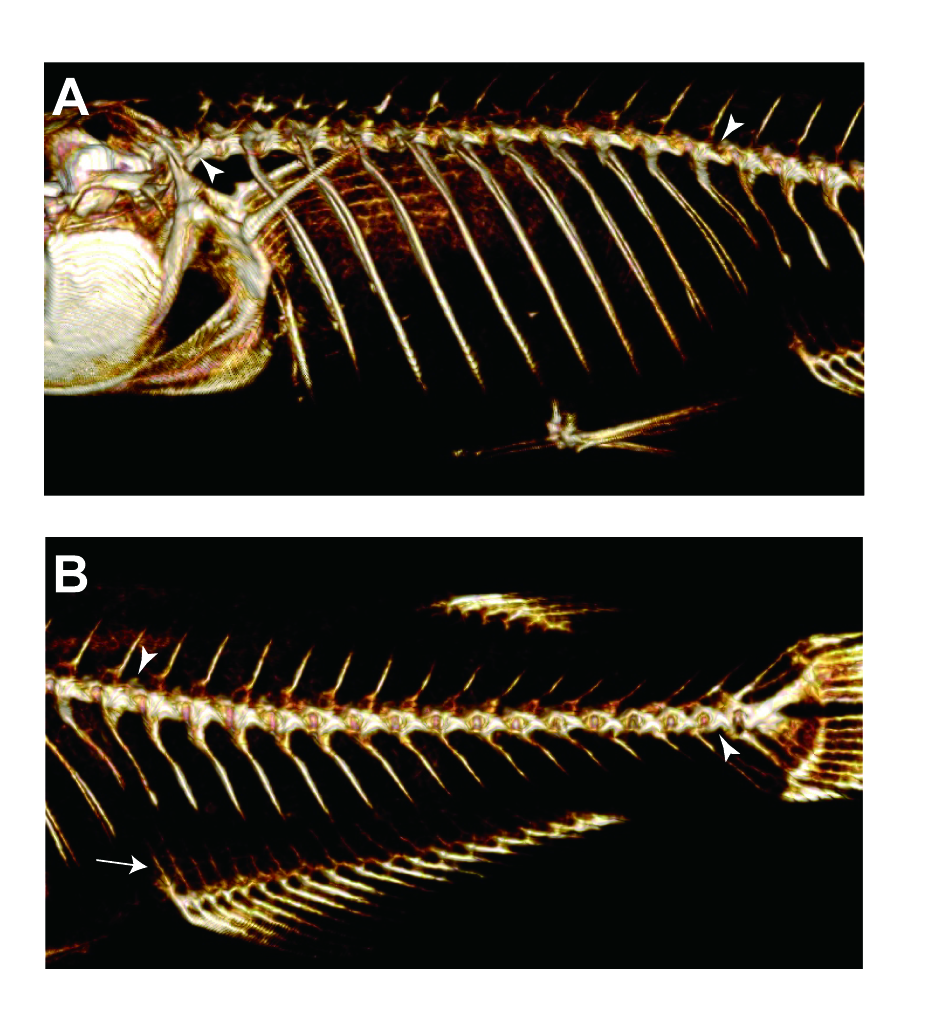

Supplement: Supplementary file 3 — Supplementary Material 3: Fig. S2. Definition of abdominal and caudal vertebrae. Arrowhead shows the first and last abdominal (A) and caudal (B) vertebrae. Arrow shows the anal pterygiophore (B). This CT reconstruction represents individual No. 6 from the WS1404_Tsuruga stock (Supplementary Table S1). [file 40851_2025_256_MOESM3_ESM.tif]

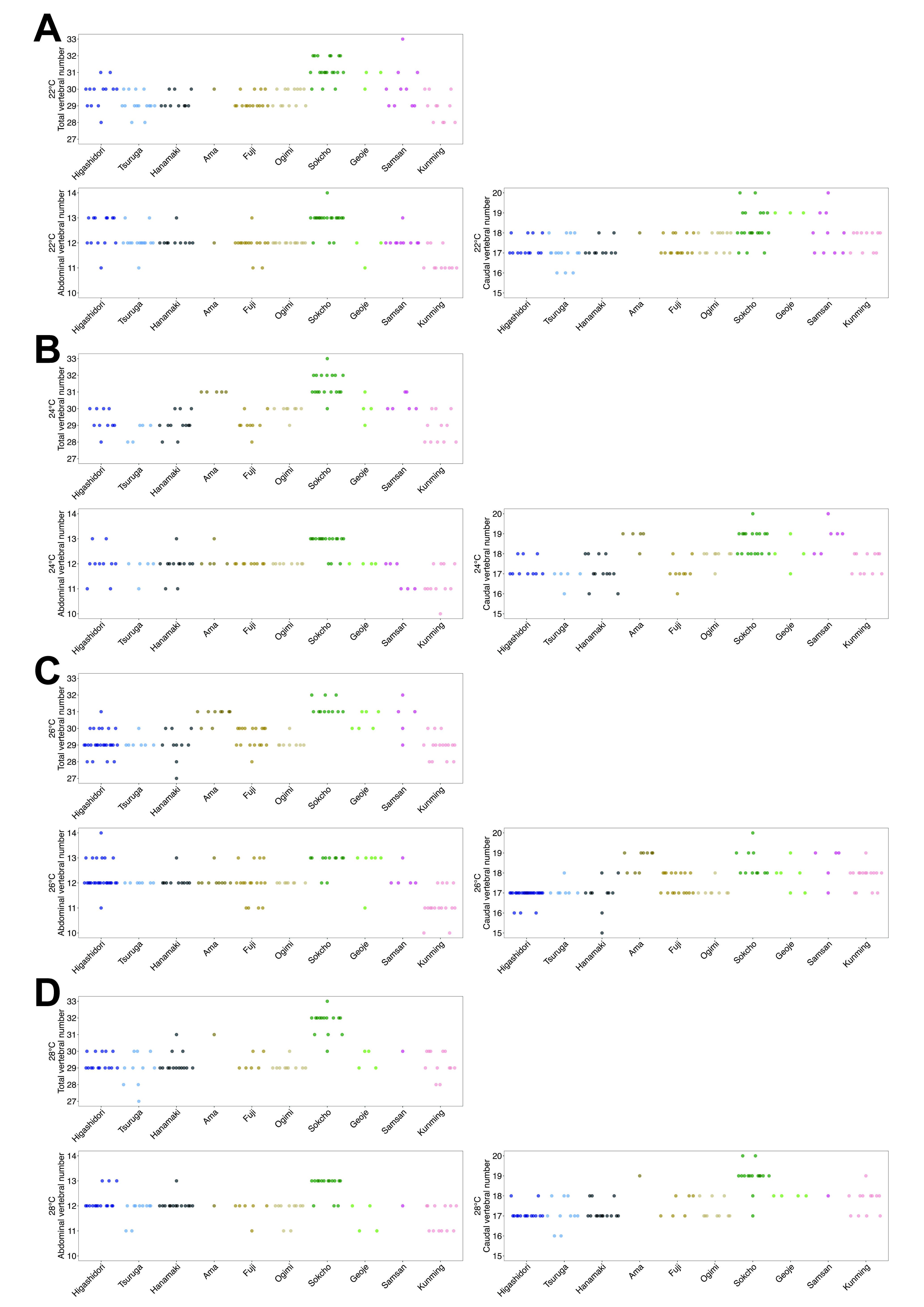

Supplement: Supplementary file 4 — Supplementary Material 4: Fig. S3. Vertebral bone numbers of each individual of 10 wild-derived stocks reared in controlled laboratory environments. Each panel indicates different water temperatures during their embryonic development; 22˚C (A), 24˚C (B), 26˚C (C), and 28˚C (D). Top, bottom left, and bottom right panels represents total, abdominal, and caudal vertebral bone numbers, respectively. [file 40851_2025_256_MOESM4_ESM.tif]
